# (2*E*,6*E*)-2,6-Difurfurylidenecyclo­hexa­none

**DOI:** 10.1107/S160053680904728X

**Published:** 2009-11-14

**Authors:** Shi-Ying Ma, Ze-Bao Zheng, Yi-Feng Sun, Zi-Ying Wang

**Affiliations:** a Department of Chemistry and Environmental Science, Taishan University, 271021 Taian, Shandong, People’s Republic of China

## Abstract

The complete mol­ecule of the title compound, C_16_H_14_O_3_, is generated by crystallographic mirror symmetry, with two C atoms and one O atom lying on the mirror plane. The mol­ecule adopts an *E* configuration about the C=C bond and the dihedral angle between the furan rings is 16.1 (2)°.

## Related literature

For general background to the use of bis­(aryl­methyl­idene)cyclo­alkanones as building blocks for the synthesis of biologically active heterocycles, see: Guilford *et al.* (1999 ▶). For related structures, see: Liu & Chen (2009 ▶); Liu (2009 ▶); Shi *et al.* (2008 ▶).
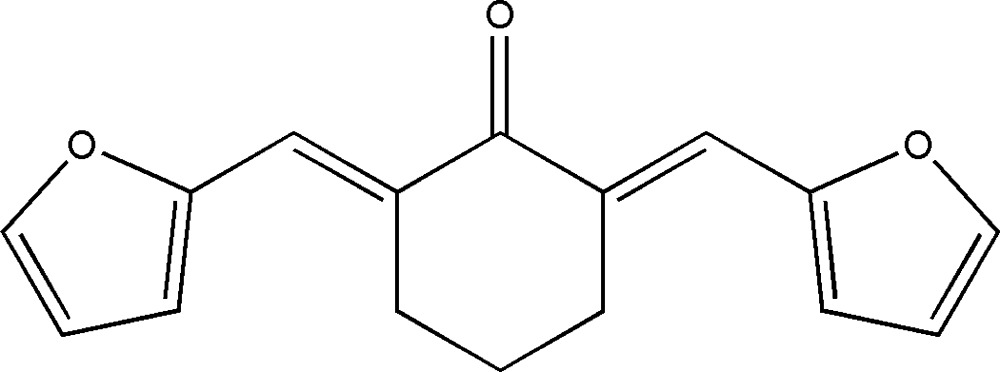


## Experimental

### 

#### Crystal data

C_16_H_14_O_3_
*M* *_r_* = 254.27
Orthorhombic, 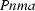

*a* = 7.7313 (11) Å
*b* = 15.658 (2) Å
*c* = 10.3388 (14) Å
*V* = 1251.5 (3) Å^3^
*Z* = 4
Mo *K*α radiation
μ = 0.09 mm^−1^
*T* = 295 K
0.15 × 0.10 × 0.06 mm

#### Data collection

Siemens SMART CCD diffractometer
Absorption correction: multi-scan (*SADABS*; Sheldrick, 1996 ▶) *T*_min_ = 0.986, *T*_max_ = 0.995
6025 measured reflections
1158 independent reflections
731 reflections with *I* > 2σ(*I*)
*R*_int_ = 0.072

#### Refinement

*R*[*F* ^2^ > 2σ(*F* ^2^)] = 0.047
*wR*(*F* ^2^) = 0.122
*S* = 1.03
1158 reflections
92 parameters
H-atom parameters constrained
Δρ_max_ = 0.17 e Å^−3^
Δρ_min_ = −0.16 e Å^−3^

### 

Data collection: *SMART* (Siemens, 1996 ▶); cell refinement: *SAINT* (Siemens, 1996 ▶); data reduction: *SAINT*; program(s) used to solve structure: *SHELXS97* (Sheldrick, 2008 ▶); program(s) used to refine structure: *SHELXL97* (Sheldrick, 2008 ▶); molecular graphics: *SHELXTL* (Sheldrick, 2008 ▶); software used to prepare material for publication: *SHELXTL*.

## Supplementary Material

Crystal structure: contains datablocks global, I. DOI: 10.1107/S160053680904728X/hb5212sup1.cif

Structure factors: contains datablocks I. DOI: 10.1107/S160053680904728X/hb5212Isup2.hkl

Additional supplementary materials:  crystallographic information; 3D view; checkCIF report

## Figures and Tables

**Fig. 1.**
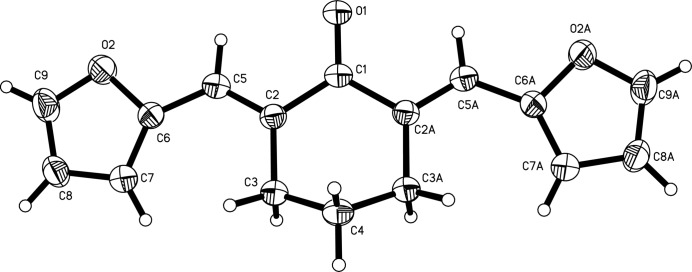
The molecular structure of (I), with displacement ellipsoids drawn at the 30% probability level. Atoms with the suffix A are generated by (x, 3/2–y, z).

## supplementary crystallographic information

### Comment

Bis(arylmethylidene)cycloalkanones are widely used as building blocks for the synthesis of biologically active heterocycles (Guilford *et al.*, 1999). In the present paper, we describe the crystal stucture of the title compound. The molecule posseses normal geometric parameters and adopts an *E*-configuration about the central olefinic bonds (Fig. 1). The cyclohexanone ring and the furan rings are alomst coplanar which allows conjugation. Similar structures have been observed in the related substituted cyclopentanone and cyclohexanone analogues reported by Liu & Chen (2009); Liu (2009); Shi *et al.* (2008).

### Experimental

Tetrabutylammonium bromide (0.5 mmol) and NaOH (10 mmol) were dissolved in the mixture of water (10 ml) and ethanol (4 ml). The solution was stirred at room temperature for 10 min, followed by dropwise addition of a mixture of furaldehyde (20 mmol) and cyclohexanone (10 mmol).The mixture was stirred at the temperature of 303 K for 2 h. When the reaction was complete, the residue was filtered. The precipitate was washed by water and recrystallized from ethyl acetate to yield yellow blocks of (I). Analysis calculated for C_16_H_14_O_3_: C 75.59, H 5.51%; found: C 75.65, H 5.46%.

### Refinement

All H-atoms were initially located in a difference Fourier map and were placed in geometrically idealized positions, with C—H = 0.93 - 0.97 Å and refined as riding with *U*_iso_(H) = 1.2*U*_eq_(C).

### Crystal data

**Table:**

C_16_H_14_O_3_	*D*_x_ = 1.349 Mg m^−^^3^
*M**_r_* = 254.27	Melting point: 417 K
Orthorhombic, *P**n**m**a*	Mo *K*α radiation, λ = 0.71073 Å
Hall symbol: -P 2ac 2n	Cell parameters from 472 reflections
*a* = 7.7313 (11) Å	θ = 2.6–19.2°
*b* = 15.658 (2) Å	µ = 0.09 mm^−^^1^
*c* = 10.3388 (14) Å	*T* = 295 K
*V* = 1251.5 (3) Å^3^	Block, yellow
*Z* = 4	0.15 × 0.10 × 0.06 mm
*F*(000) = 536	

### Data collection

**Table:**

Siemens SMART CCD diffractometer	1158 independent reflections
Radiation source: fine-focus sealed tube	731 reflections with *I* > 2σ(*I*)
graphite	*R*_int_ = 0.072
ω scans	θ_max_ = 25.1°, θ_min_ = 2.4°
Absorption correction: multi-scan (*SADABS*; Sheldrick, 1996)	*h* = −9→9
*T*_min_ = 0.986, *T*_max_ = 0.995	*k* = −15→18
6025 measured reflections	*l* = −12→9

### Refinement

**Table:**

Refinement on *F*^2^	Secondary atom site location: difference Fourier map
Least-squares matrix: full	Hydrogen site location: inferred from neighbouring sites
*R*[*F*^2^ > 2σ(*F*^2^)] = 0.047	H-atom parameters constrained
*wR*(*F*^2^) = 0.122	*w* = 1/[σ^2^(*F*_o_^2^) + (0.0454*P*)^2^ + 0.2592*P*] where *P* = (*F*_o_^2^ + 2*F*_c_^2^)/3
*S* = 1.03	(Δ/σ)_max_ < 0.001
1158 reflections	Δρ_max_ = 0.17 e Å^−^^3^
92 parameters	Δρ_min_ = −0.16 e Å^−^^3^
0 restraints	Extinction correction: *SHELXL97* (Sheldrick, 2008), Fc^*^=kFc[1+0.001xFc^2^λ^3^/sin(2θ)]^-1/4^
Primary atom site location: structure-invariant direct methods	Extinction coefficient: 0.017 (2)

### Special details

**Table:**

Geometry. All e.s.d.'s (except the e.s.d. in the dihedral angle between two l.s. planes) are estimated using the full covariance matrix. The cell e.s.d.'s are taken into account individually in the estimation of e.s.d.'s in distances, angles and torsion angles; correlations between e.s.d.'s in cell parameters are only used when they are defined by crystal symmetry. An approximate (isotropic) treatment of cell e.s.d.'s is used for estimating e.s.d.'s involving l.s. planes.
Refinement. Refinement of *F*^2^ against ALL reflections. The weighted *R*-factor *wR* and goodness of fit *S* are based on *F*^2^, conventional *R*-factors *R* are based on *F*, with *F* set to zero for negative *F*^2^. The threshold expression of *F*^2^ > σ(*F*^2^) is used only for calculating *R*-factors(gt) *etc*. and is not relevant to the choice of reflections for refinement. *R*-factors based on *F*^2^ are statistically about twice as large as those based on *F*, and *R*- factors based on ALL data will be even larger.

### Fractional atomic coordinates and isotropic or equivalent isotropic displacement parameters (Å^2^)

**Table:**

	*x*	*y*	*z*	*U*_iso_*/*U*_eq_	
O1	0.0245 (3)	0.7500	0.0550 (2)	0.0606 (7)	
O2	0.1187 (2)	0.44953 (11)	0.12636 (15)	0.0689 (6)	
C1	0.0568 (4)	0.7500	0.1720 (3)	0.0461 (8)	
C2	0.0806 (3)	0.66875 (14)	0.24340 (19)	0.0434 (6)	
C3	0.0950 (3)	0.67029 (14)	0.3886 (2)	0.0509 (7)	
H3A	0.2160	0.6668	0.4129	0.061*	
H3B	0.0367	0.6206	0.4239	0.061*	
C4	0.0172 (4)	0.7500	0.4467 (3)	0.0527 (9)	
H4A	0.0366	0.7500	0.5394	0.063*	
H4B	−0.1067	0.7500	0.4319	0.063*	
C5	0.0954 (3)	0.59655 (14)	0.1739 (2)	0.0494 (6)	
H5	0.0794	0.6022	0.0852	0.059*	
C6	0.1324 (3)	0.51243 (15)	0.2189 (2)	0.0497 (7)	
C7	0.1845 (4)	0.47563 (15)	0.3310 (2)	0.0602 (7)	
H7	0.2048	0.5036	0.4089	0.072*	
C8	0.2024 (4)	0.38729 (16)	0.3081 (2)	0.0646 (8)	
H8	0.2366	0.3460	0.3674	0.078*	
C9	0.1606 (4)	0.37468 (17)	0.1841 (3)	0.0729 (9)	
H9	0.1603	0.3218	0.1432	0.087*	

### Atomic displacement parameters (Å^2^)

**Table:**

	*U*^11^	*U*^22^	*U*^33^	*U*^12^	*U*^13^	*U*^23^
O1	0.0879 (19)	0.0532 (15)	0.0406 (13)	0.000	−0.0145 (12)	0.000
O2	0.1102 (16)	0.0485 (11)	0.0481 (10)	0.0097 (10)	0.0021 (10)	−0.0052 (8)
C1	0.051 (2)	0.051 (2)	0.0365 (17)	0.000	−0.0044 (15)	0.000
C2	0.0445 (14)	0.0456 (14)	0.0402 (12)	−0.0018 (11)	−0.0005 (10)	−0.0007 (11)
C3	0.0656 (17)	0.0479 (14)	0.0391 (12)	−0.0026 (13)	−0.0014 (11)	0.0010 (11)
C4	0.061 (2)	0.059 (2)	0.0387 (17)	0.000	0.0036 (16)	0.000
C5	0.0538 (15)	0.0524 (16)	0.0421 (13)	−0.0002 (12)	−0.0027 (11)	0.0021 (11)
C6	0.0596 (17)	0.0451 (14)	0.0445 (13)	−0.0001 (12)	0.0003 (11)	−0.0024 (11)
C7	0.0767 (19)	0.0525 (16)	0.0513 (14)	−0.0008 (14)	−0.0073 (13)	−0.0004 (12)
C8	0.081 (2)	0.0479 (16)	0.0650 (17)	0.0033 (14)	0.0018 (15)	0.0110 (13)
C9	0.109 (2)	0.0432 (16)	0.0669 (18)	0.0100 (16)	0.0138 (17)	0.0018 (14)

### Geometric parameters (Å, °)

**Table:**

O1—C1	1.235 (3)	C4—H4A	0.9700
O2—C9	1.355 (3)	C4—H4B	0.9700
O2—C6	1.377 (2)	C5—C6	1.426 (3)
C1—C2^i^	1.482 (3)	C5—H5	0.9300
C1—C2	1.482 (3)	C6—C7	1.355 (3)
C2—C5	1.344 (3)	C7—C8	1.410 (3)
C2—C3	1.506 (3)	C7—H7	0.9300
C3—C4	1.510 (3)	C8—C9	1.336 (3)
C3—H3A	0.9700	C8—H8	0.9300
C3—H3B	0.9700	C9—H9	0.9300
C4—C3^i^	1.510 (3)		
			
C9—O2—C6	107.08 (19)	C3^i^—C4—H4B	109.3
O1—C1—C2^i^	120.85 (13)	H4A—C4—H4B	108.0
O1—C1—C2	120.85 (13)	C2—C5—C6	128.3 (2)
C2^i^—C1—C2	118.2 (3)	C2—C5—H5	115.9
C5—C2—C1	117.8 (2)	C6—C5—H5	115.9
C5—C2—C3	122.7 (2)	C7—C6—O2	108.2 (2)
C1—C2—C3	119.5 (2)	C7—C6—C5	137.0 (2)
C2—C3—C4	112.3 (2)	O2—C6—C5	114.73 (19)
C2—C3—H3A	109.1	C6—C7—C8	107.6 (2)
C4—C3—H3A	109.1	C6—C7—H7	126.2
C2—C3—H3B	109.1	C8—C7—H7	126.2
C4—C3—H3B	109.1	C9—C8—C7	106.4 (2)
H3A—C3—H3B	107.9	C9—C8—H8	126.8
C3—C4—C3^i^	111.5 (3)	C7—C8—H8	126.8
C3—C4—H4A	109.3	C8—C9—O2	110.7 (2)
C3^i^—C4—H4A	109.3	C8—C9—H9	124.7
C3—C4—H4B	109.3	O2—C9—H9	124.7
			
O1—C1—C2—C5	11.1 (4)	C9—O2—C6—C7	0.9 (3)
C2^i^—C1—C2—C5	−166.16 (18)	C9—O2—C6—C5	179.0 (2)
O1—C1—C2—C3	−171.5 (3)	C2—C5—C6—C7	−10.1 (5)
C2^i^—C1—C2—C3	11.2 (4)	C2—C5—C6—O2	172.5 (2)
C5—C2—C3—C4	−161.0 (2)	O2—C6—C7—C8	−0.5 (3)
C1—C2—C3—C4	21.8 (3)	C5—C6—C7—C8	−178.0 (3)
C2—C3—C4—C3^i^	−55.1 (3)	C6—C7—C8—C9	0.0 (3)
C1—C2—C5—C6	174.6 (2)	C7—C8—C9—O2	0.6 (3)
C3—C2—C5—C6	−2.7 (4)	C6—O2—C9—C8	−0.9 (3)

Symmetry codes: (i) *x*, −*y*+3/2, *z*.
